# Electrochemical lysis of an intraocular tumour using a combination of electrode placements

**DOI:** 10.3332/ecancer.2012.272

**Published:** 2012-10-08

**Authors:** Y Belyy, A Tereshchenko, A Shatskih

**Affiliations:** The Interdisciplinary Scientific and Technological Complex (ISTC) Eye Microsurgery, Kaluga, Russia

## Abstract

**The objective** of this experimental study was to develop a new combination technique for electrode placement and the histomorphological evaluation of its effectiveness for the electrochemical lysis of large intraocular tumours.

**Materials and methods:**

The ECL was conducted on two freshly enucleated eyes containing large tumours, with maximal prominence of 11 and 12 mm and maximal base diameter of 16 and 19 mm, respectively. The ECL was carried out using an ECU-300 (Soring, Germany) apparatus generating an electrochemical charge of 30-35 K. In the course of the ECL procedure we used a new original combination technique of electrode placement, i.e., the anode was a surface electrode and the cathode was an intrastromal electrode. The anode had an original design.

**Results:**

A greyscale B-scan performed after the ECL completion showed decreased echogenicity and heterogeneity of the echo-structure of the tumour. According to the bioimpedancemetry data, the average duration of the ECL session was from 20 to 30 minutes depending on the tumour size. The results of pathomorphological examination performed after the ECL on two freshly enucleated eyes appeared to be similar. Thus, in both cases after lysis the eyeball did not change its size or shape. In both cases the tumour originated from the choroid plexus and showed subtotal necrosis. There was a pronounced boundary between the intact and electrochemically damaged tumour regions which attests to the local effect of the ECL restricted to the electrode placement area only.

**Discussion:**

The growing interest in the ECL procedure is due not only to its availability and low cost but mainly to its real clinical effect demonstrated in numerous publications. The absence of a developed ECL technology for the treatment of intraocular tumors, and, hence, reports on its clinical effectiveness, gave us the impetus to conduct this study.

The proposed ECL method is promising and can be considered as optional for the organ-sparing treatment of large-sized intraocular tumours. Further optimization of the ECL parameters, as well as the development of sets of surface and intrastromal electrodes for different types of tumours, is required.

## Introduction

Choroidal melanoma (CM) is the most common primary malignant intraocular tumour that accounts for nearly 80% of all choroid plexus tumours. Owing to the high risk of metastases (3–16%) [1–4], the CM is associated with a very poor prognosis which renders CM both a vision- and life-threatening condition. In Russia, the CM prevalence rate was estimated to be 6–8 cases per one million people.

Nowadays, the preferred concept in ocular oncology for CM treatment is the use of organ-sparing methods which basically require radical surgery against the tumour while causing minimal damage to surrounding normal tissues.

Currently, the range of organ-sparing methods available for CM treatment is wide enough and includes laser photocoagulation, brachytherapy, cryodestruction, transpupillary thermotherapy, photodynamic therapy, surgical resection of the tumour (en block resection), etc. [4].

The possibility of the use of organ-sparing treatment of CM will largely depend on the tumour size (its maximal base diameter and prominence should not exceed 13–14 and 6.5 mm, respectively) and location (post-equatorial). Enucleation is performed to remove large-sized eye tumours.

In view of the above, the development of new minimally invasive and organ-sparing methods for the treatment of large CMs (usually treated by enucleation) is becoming a topical issue.

A good example of the organ-sparing trend in oncology is the electrochemical lysis (ECL) method which induces destructive chemical reactions occurring during direct current flow between two bipolar electrodes introduced into the tumour (HCl is produced at the anode and NaOH at the cathode), that subsequently results in tissue coagulation and colliquative necrosis around the electrodes.

The ECL method has been fairly successfully used for the treatment of breast cancer, hepatic carcinoma, and tumour metastases in the liver, benign prostatic hyperplasia, cancer of oesophagus, lungs, pancreas, and skin [5–11].

In general oncology, the standard ECL technique involves a parallel introduction of two or more needle electrodes into the tumour. Using a similar approach in ocular oncology, the electrodes should be introduced in the intraocular tumour ***transsclerally in the zone of projection of the tumour base onto the sclera. In order to obtain an adequate necrosis of large tumours, three or more electrodes must be introduced intrastromally and correctly positioned under ultrasound control (grey scale B-scan) which is fraught with risks and several complications, such as iatrogenic retina tear, hemophthalmos, subretinal and subchoroidal haemorrhages, etc.

Therefore, the problems with electrode placement and inability to predict the optimal electric field’s effect on the tumour make the search for new approaches to ECL application in ocular oncology a topical undertaking.

The objective of this experimental study was to develop a new combination technique for electrode placement and the histomorphological evaluation of its effectiveness for the ECL of the large-sized intraocular tumours.

## Materials and methods

The ECL was conducted on two freshly enucleated eyes containing the large-sized tumours, with maximal prominence of 11 and 12 mm and maximal base diameter of 16 and 19 mm, respectively.

**Figure 1:**
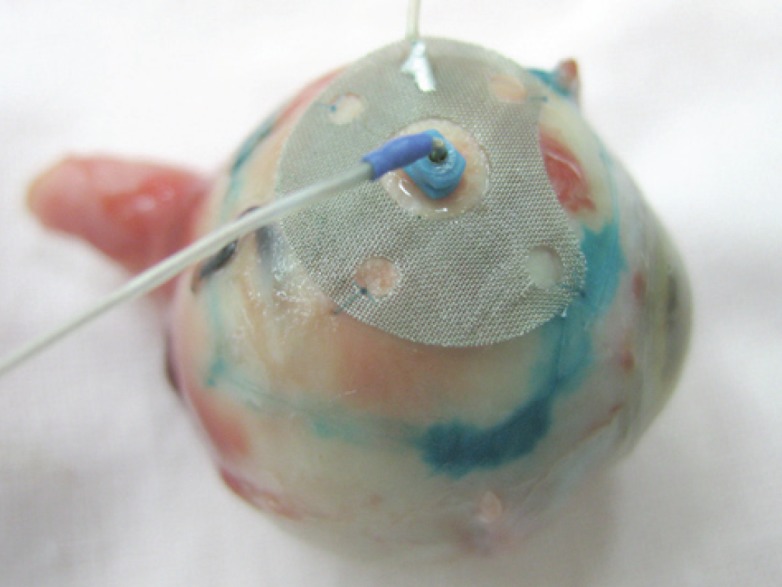
Combination placement of electrodes for the ECL.

The ECL was carried out using the “ECU-300” (Soring, Germany) apparatus generating an electrochemical charge of 30–35 K. In the course of the ECL procedure, we used a new original combination technique of electrode placement, i.e., the anode was a surface electrode and the cathode was an intrastromal electrode. The anode had an original design: it was made of the platinum wire to form a round mesh of 9 mm in diameter and with a hole in the centre. The cathode was a needle electrode made of 0.5 mm platinum wire (Figure 1).

At the ECL preparation step, we determined the boundaries of the tumour base projection onto the sclera by their marking with 1% water– alcohol solution of brilliant green. To perform the ECL, the anode was applied onto the sclera within previously marked boundaries of the tumour base and stitched with two interrupted sutures. The cathode was introduced perpendicularly to the sclera into the centre of the tumour base through the hole in the anode electrode. To introduce the cathode into the tumour, a trocar with a screw-type length adjustment and a 25-G cannula were used.

The length of trocar was adjusted to accommodate the length of the extra-scleral part of the 25-G cannula + sclera thickness + electrode penetration depth into the tumour. Then, sclerotomy was performed using the trocar inserted into the lumen of the cannula, by inserting it to its full depth into the tumour perpendicularly to the sclera followed by trocar withdrawal from the lumen of the cannula and its replacement with the electrode of a predetermined length.

The depth to which the electrode was inserted into the tumour had been determined in advance based on the results of previous ultrasonic scanning (grey scale B-scan) by subtracting 1.5–2 mm from the value of the maximum prominence in the centre of the tumour. The length of the electrode active part was calculated in the same manner as the trocar length (RF patent for an invention no. 2375020, priority of 12.08.2008; RF patent for an invention no. 2347548, priority of 17.10.2007).

The active positioning of the intrastromal electrode was carried out in the course of surgery under the trans-corneal and trans-scleral ultrasound control using a 10-MHz probe of the Ultrascan (Alcon, Fort Worth, TX) apparatus.

**Figure 2:**
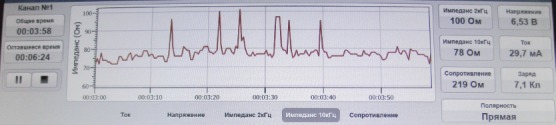
Tissue impedance changes in the course of ECL (real-time recording).

To evaluate the ECL effectiveness, we used the bioimpedancemetry method which is the measurement of total electrical resistance of tumour tissue placed between two electrodes in the direct electric field of variable frequency. Multiple impedance measurements of lysed tissue in the course of ECL were performed using an experimental setup at 2 and 10 kHz. To this end, the ECL procedure was interrupted every 3 min for 1–2 s. The same electrodes were used for both ECL and bioimpedancemetry. Impedance measurement (*Z*) was conducted in an automated mode, and the software produced the chart of changes of tissue resistance in real-time (Figure 2). Once the impedance values (*Z*) flattened out and appeared to be hardly affected by time, it was an indication for completion of the ECL procedure.

Before starting the lysis, the cannula was filled with ca. 0.1–0.2 ml of BSS solution, and surface electrode was rinsed in order to reduce resistance between the electrodes. The electrode polarity was not changed throughout the procedure. In the course of ECL, there was an increase of the intraocular pressure which was lowered by removal of the tumour breakdown products. To this end, the course of ECL was interrupted, the intrastromal electrode was withdrawn, and the tip of the 25-G vitreotom was introduced through the cannula to the accurately set depth depending on the tumour prominence. After the completion of this step, the vitreotom was withdrawn, the volume of tissue removed was replenished with BSS, the electrode was inserted, and the ECL procedure was resumed using the same parameter settings. At 2–3 min before the ECL completion and without interrupting its course, the plastic isolating cannula was removed from the sclera in order to rule out the presence of tumour regions unaffected by lysis. Upon the ECL completion, the surface and intrastromal electrodes were removed along with the cannula. The sclerotomy wound was not sutured.

The pathomorphological examinations were performed to determine the extent of the post-ECL damage to the intraocular tumour. To this end, the enucleated eye balls were fixed in neutral formaldehyde solution, rinsed with tap water, dehydrated in ascending grades of alcohol and embedded in paraffin, followed by serial microtome sectioning and haematoxylin-eosin staining for histological examination.

## Results

The grey scale B-scan performed after the ECL completion showed decreased echogenicity and heterogeneity of the echo-structure of the tumour. However, the presence of numerous gas bubbles within the tumour structure complicated the ultrasonic imaging.

According to the bioimpedancemetry data, the average duration of the ECL session was from 20 to 30 minutes depending on the tumour size.

The results of pathomorphological examination performed after the ECL on two freshly enucleated eyes appeared to be similar. Thus, in both cases after lysis the eyeball did not change its size or shape. The area of intervention was located within the tumour projection occupying the area of 63.6 mm2 equal to meshed electrode (anode). In the centre of the area there was a canal resulted from the insertion of the intrastromal electrode (cathode), its opening containing dark fluid expelled after the electrode withdrawal. When the eyeball was cut open, the impurity-free liquid of the vitreous body leaked out through the base of the tumour. The location of eye integuments and tumour was in accord with the results of clinical and instrumental examination. On section, the tumour is dark and has small slit-shaped spaces through which a slightly frothy, gel-like liquid, with admixture of reddish-brown, blood-tinged fluid, is oozing.

In both cases the tumour was originated from the choroid plexus and showed subtotal necrosis.

For the sake of convenience of description of the ECL-induced tumour morphological changes, the tumour was conditionally divided into three parts: the apex, middle part and periscleral part.

**Figure 3:**
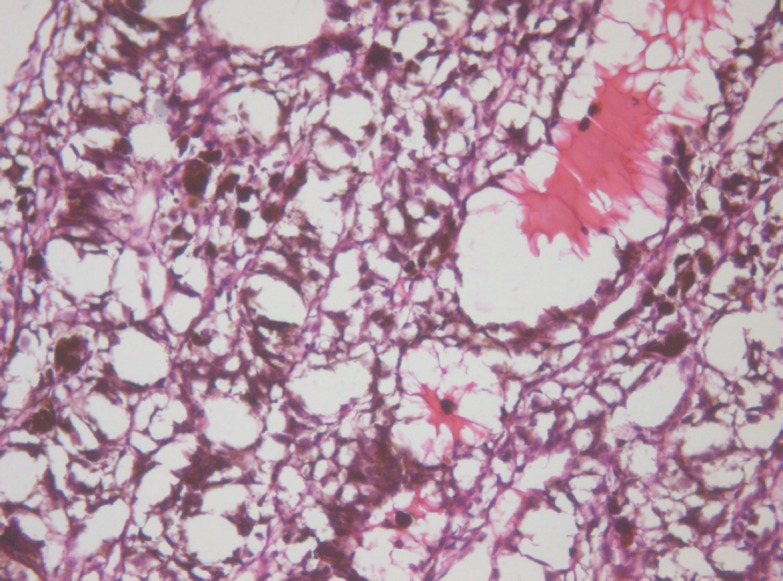
Picture of choroidal melanoma after the ECL procedure. Destruction zone: destruction of vascular walls, tumour cell fragmentation, and destruction of cell nuclei (caryopicnosis, caryorexis), pigment condensation. Hematoxylin-eosin staining, magnification ×200.

The apex part showed necrosis with cell fragmentation, nucleus contractions and stiffing (caryopicnosis) and nucleus fragmentations (caryorexis), pigment condensation, appearance of slit-shaped spaces in place of the vessels filled with lysed blood and gaps along the contour of the palisade structures (Figure 3).

**Figure 4:**
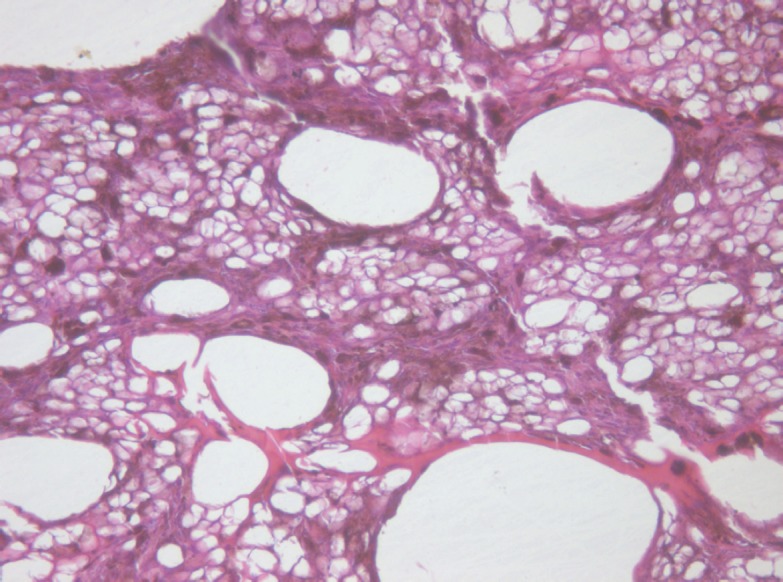
Picture of choroidal melanoma after the ECL procedure. Destruction zone: gaps (cavities with liquid content) in place of vessels and tumour cells. Hematoxylin-eosin staining, magnification ×200.

The middle part demonstrated total cell necrosis. The contours of the gaps resemble those of individual cells and lumens of destroyed vessels (Figure 4). The septa between the cavities are represented by remnants of the minimal stromal component of the tumour, pigment granules and cell-free debris compressed because of pressure of the liquid accumulated in the gaps.

**Figure 5:**
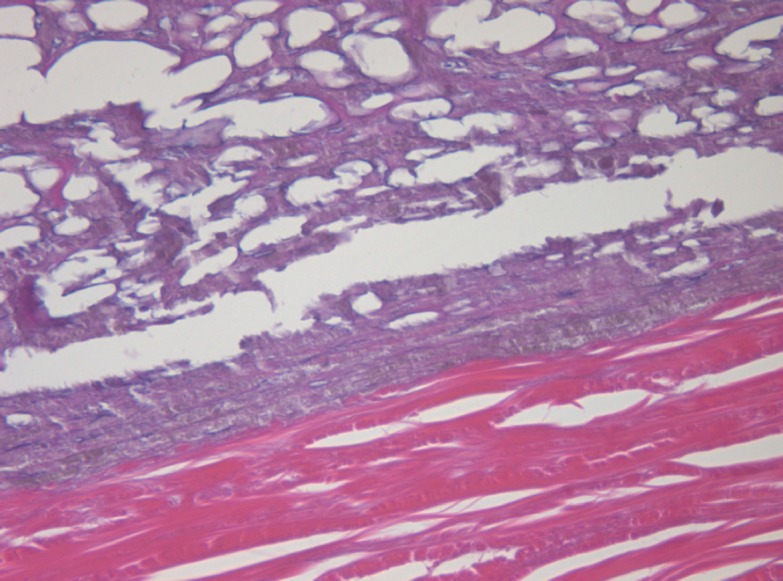
Picture of choroidal melanoma after the ECL procedure. Periscleral zone of destruction: gaps in place of tumour cells, depigmentation. Hematoxylin-eosin staining, magnification ×400.

In the periscleral part of the tumour located next to the cathode canal the morphological picture resembled that of the middle part except for the smaller size cavities, owing to the absence of large vessels in this part of the tumour (Figure 5).

**Figure 6:**
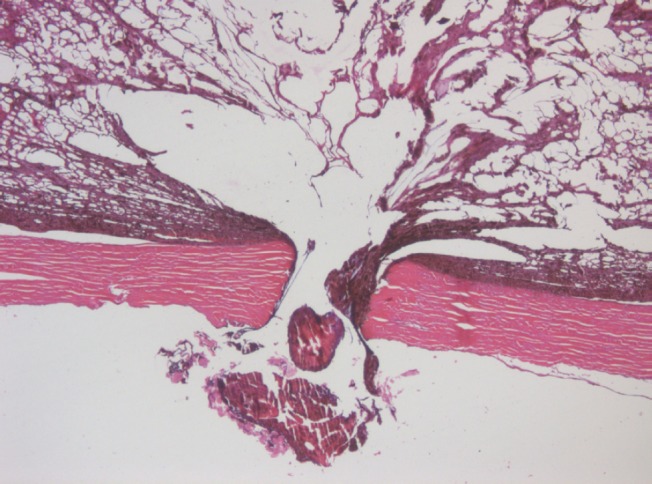
Picture of choroidal melanoma after the ECL procedure. Electrode canal in the sclera is filled with pigmented cell-free debris with an admixture of lysed blood. Hematoxylin-eosin staining, magnification ×50.

The lumen of the canal formed after withdrawal of the intra-scleral cathode electrode) is filled with pigmented debris with an admixture of lysed blood (Figure 6).

Given that the area of the surface electrode is smaller than the tumour base area, some periscleral spots of the intact tumour tissue have been found. These spots were characteristic for CM, i.e., they were intensively pigmented and composed mainly of spindle cell type A melanocytes which showed moderate polymorphism and minimal infiltration of the inner layers of the sclera.

**Figure 7:**
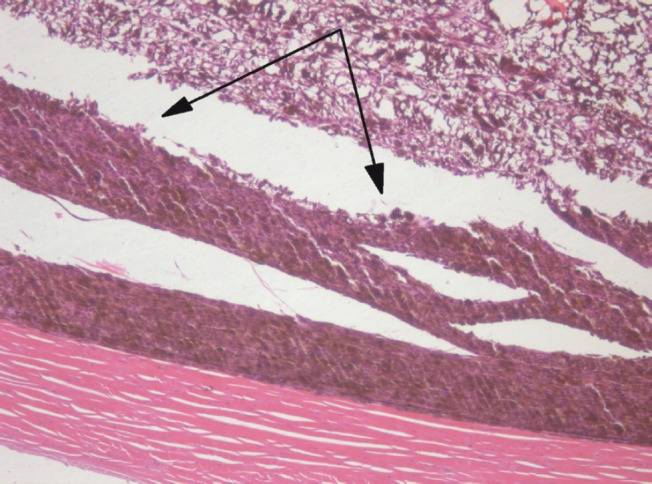
Picture of choroidal melanoma after the ECL procedure. Periscleral spots with preserved intact tumour tissue (*arrowed*). Hematoxylin-eosin staining, magnification ×100.

There was a pronounced boundary between the intact and electrochemically damaged tumour regions (Figure 7) which attests to local effect of the ECL restricted to the electrode placement area only.

## Discussion

The growing interest in the ECL procedure is due not only to its availability and low cost but mainly to its real clinical effect demonstrated in numerous publications [5, 7–14].

The absence of developed ECL technology for treatment of intraocular tumours, and, hence, reports on its clinical effectiveness, gave us the impetus to conduct this study.

We set out to develop a new method making use of combination of the surface and intrastromal electrodes, as well as their original placement, which hitherto has never been described. To evaluate the ECL effectiveness, we performed the bioimpedancemetry of the ECL-treated tissues and morphological examinations of the large-sized intraocular tumours. The experiment was aimed at developing an integrated and manageable ECL method based on unbiased impedancemetry measurements, thus allowing the evaluation of its dynamics and determination of the point of its completion.

The direct current flow between the electrodes results in tissue devitalization owing to its electrolysis. In the course of the ECL procedure the resistance of tissue (Z) positioned between the electrodes drops indicating the occurrence of tissue necrosis. The registration of therapeutic effect (i.e., tumour necrosis and, hence, completion of the ECL procedure) is based on the impedancemetry measurements which are consistent and little changeable with time.

The histological picture of the ECL-induced CM necrosis demonstrates different patterns of tumour destruction, primarily of its vessels, around each electrode depending on their polarity.

Under the cathode electrode a pronounced vasodilatation and engorgement of large vessels occur accompanied by the capillary wall destruction and extensive hemorrhages into the necrotized tissue due to an increased turgor pressure caused by the electro-osmotic fluid flow. On the anode side the capillary reaction was only slightly noticeable. 

Therefore, the electromagnetic field effects in biological tissues are due to the obstruction of the microvascular bed. In the cathode region the capillaries are blocked owing to the electro-osmotic fluid flow, and in the anode region the pathological changes occur due to the micro-thrombotic events. Owing to the remote placement of the electromagnetic field source the final area of the ECL-induced tissue lesions must exceed the total area of primary necrosis.

The presence of the periscleral sites of intact tumour separated by a pronounced boundary from the electrochemically damaged tumour spots indicates to the importance of the accurate placement of surface electrode, as well as its selection, taking into account the size of tumour base projection onto the sclera and avoiding the use of electrodes with smaller contact area.

One of the particular features of ECL treatment of the intraocular tumours is the increased intraocular pressure occurred in the course of the ECL procedure. This is due to active gas bubble formation within the tumour and hampered evacuation of liquid debris via the electrode canal. Therefore, the removal of cell-free products of tumour necrosis using the vitreotom secures the maintenance of the original level of intraocular pressure, does not interfere with the ECL procedure and enables process stability owing to the replenishment of evacuated volume with BSS saline.

The updated pattern of the electrode placement geometry using surface extrascleral and intrastromal electrodes and adherence to correct polarity while placing electrode in the tumour, opens up new horizons in achieving total intraocular tumour destruction. The design feature of this ECL technique is an individual selection of surface electrode which covers the whole tumour base over its projection onto the sclera, while a centrally positioned hole enables to introduce the intrastromal electrode to any depth allowing its placement maximally close to the tumour apex and preventing electrode dislocation within this position.

When setting the parameters of ECL, we were guided by the charge value which would knowingly cause necrosis of a given tumour volume. This value was experimentally established to be 30 K per 1 cm3 of tumour tissue, and the increase of charge above this value does not practically cause the expansion of necrosis zone [15, 16]. However, all previous experimental and clinical studies on working out the main ECL parameters were carried out using the parallel electrode placement in the tumour. Our used experimental parameters included the combination of electrode placement on freshly enucleated eyes, charge value of 30 K and duration of the ECL procedure of 20 to 30 minutes, and likewise provided the achievement of total intraocular tumour necrosis. Nonetheless, in case of large-sized and non-homogeneous tumours the lysis parameters (e.g., current and voltage) can be adjusted, and, hence, it warrants further experimental and clinical investigations.

Further modifications, refinement and customizing of the ECL method to address the specific needs of ophthalmologic oncology will allow the pre-modelling of tumour necrosis patterns aided by using the specifically designed software, and thus will help to achieve higher treatment effectiveness. Working out of the objective method of real-time assessment of pathologic changes occurring within the tumour, e.g., by measuring active and reactive tissue resistance by means of bio impedancemetry, provides the effective control and regulation of the ECL procedure.

Also, the above method offers additional benefits in investigating tumour morphology and growth pattern, as the fine-needle aspiration biopsy of the intraocular tumour can be performed simultaneously with electrode insertion.

Undoubtedly, the size of the intraocular tumour destruction occurred after clinical use of the ECL method will depend not only on the parameters of the procedure but also on the duration of the post-ECL time period. To elucidate these issues, the insightful information from clinical trials will be required.

## Conclusion

Our experimental study indicates that the new ECL method employing the original combination of electrode placement combines minimal tissue injury and complete tumour destruction under the area of electrode application. The use of surface electrode enables to direct and evoke destruction of the whole tumour base area.

The combination of surface and intrastromal electrodes allows the minimal damage to the scleral integrity in the site of tumour base projection. Further experiments dealing with variations in depth of the intrastromal electrode insertion and the amount of current, coupled with bioimpedancemetry, will enable the regulation of morphological changes within the tumour.

The proposed ECL method is promising and can be considered as optional for the organ-sparing treatment of large-sized intraocular tumours. Further optimization of the ECL parameters, as well as the development of sets of surface and intrastromal electrodes for different types of tumours, is required.
